# Minocycline-Induced Acute Pancreatitis With Cyst Formation in a Young Man

**DOI:** 10.7759/cureus.72472

**Published:** 2024-10-27

**Authors:** Keita Odaka, Katsunori Sekine, Tomoyuki Yada, Masaaki Mino, Naomi Uemura

**Affiliations:** 1 Gastroenterology and Hepatology, Kohnodai Hospital, National Center for Global Health and Medicine, Ichikawa City, JPN

**Keywords:** acute pancreatitis, drug-induced acute pancreatitis (diap), minocycline, pancreatic cyst, severe pancreatitis

## Abstract

An 18-year-old male, diagnosed with urethritis and treated with minocycline for six days, presented to our hospital complaining of abdominal pain and nausea. Blood tests and contrast-enhanced computed tomography (CT) showed severe acute pancreatitis. Based on his medical history, blood tests, and imaging studies, common etiologies of acute pancreatitis were excluded, including alcohol intake, anatomical abnormalities of the pancreas and biliary tract, stones, malignancy, autoimmune diseases, and lipid abnormalities. The patient was diagnosed with drug-induced acute pancreatitis due to minocycline.

Upon admission, minocycline was discontinued, and the patient was treated symptomatically. His symptoms improved steadily, and he was discharged on day 14 of hospitalization. A follow-up CT scan on day 8 of hospitalization revealed a 4-cm cyst at the pancreatic tail; however, since the patient was asymptomatic, he was monitored with imaging studies. The cyst gradually shrank and was no longer visible on a CT scan 12 weeks after discharge.

Most cases of drug-induced acute pancreatitis caused by tetracycline have been reported as mild to moderate, and there are no documented cases of severe pancreatitis with cyst formation in the literature. This case illustrates that minocycline-induced acute pancreatitis can lead to severe pancreatitis and cyst formation, warranting careful use.

## Introduction

Acute pancreatitis (AP) is a benign but sometimes potentially fatal disease [1]. Most cases are alcohol-induced or cholelithiasis [1], whereas drug-induced AP (DIAP) is very rare (<2%) [2]. Most DIAP cases are mild or moderate, and their prognosis is relatively good. However, severe or fatal cases can occur [3]. Failure to identify the causative agent can lead to AP flare-ups, severe delays, and adverse outcomes [1]. The diagnosis of DIAP is difficult for clinicians and requires ruling out more common etiologies of AP, such as alcohol abuse, cholelithiasis, or familial lipidosis. Drugs that may cause AP should be discontinued or replaced with alternative agents.

The World Health Organization database lists over 500 drugs with the potential to cause DIAP [4]. Minocycline is a commonly used antimicrobial agent, with pancreatitis as one of its side effects. We have found only a few case reports of minocycline-induced severe AP, we report a case of severe DIAP with cyst formation in a patient receiving minocycline.

## Case presentation

An 18-year-old Japanese man with no significant medical history was diagnosed with urethritis and started on minocycline 200 mg/day. After six days of the antibiotic treatment, he developed severe abdominal pain on his left side with vomiting. He denied using any other new medication or over-the-counter substance, drinking alcohol, recent consumption of copious meals, or having a history of abdominal trauma. He had no medical history of biliary lithiasis, pancreatic diseases, or hyperlipidemia, and denied any family history of those. Additionally, he had not traveled recently. Physical examination showed that he was hemodynamically stable and afebrile, with only tenderness on the left side of his abdomen observed on palpation.

**Figure 1:**
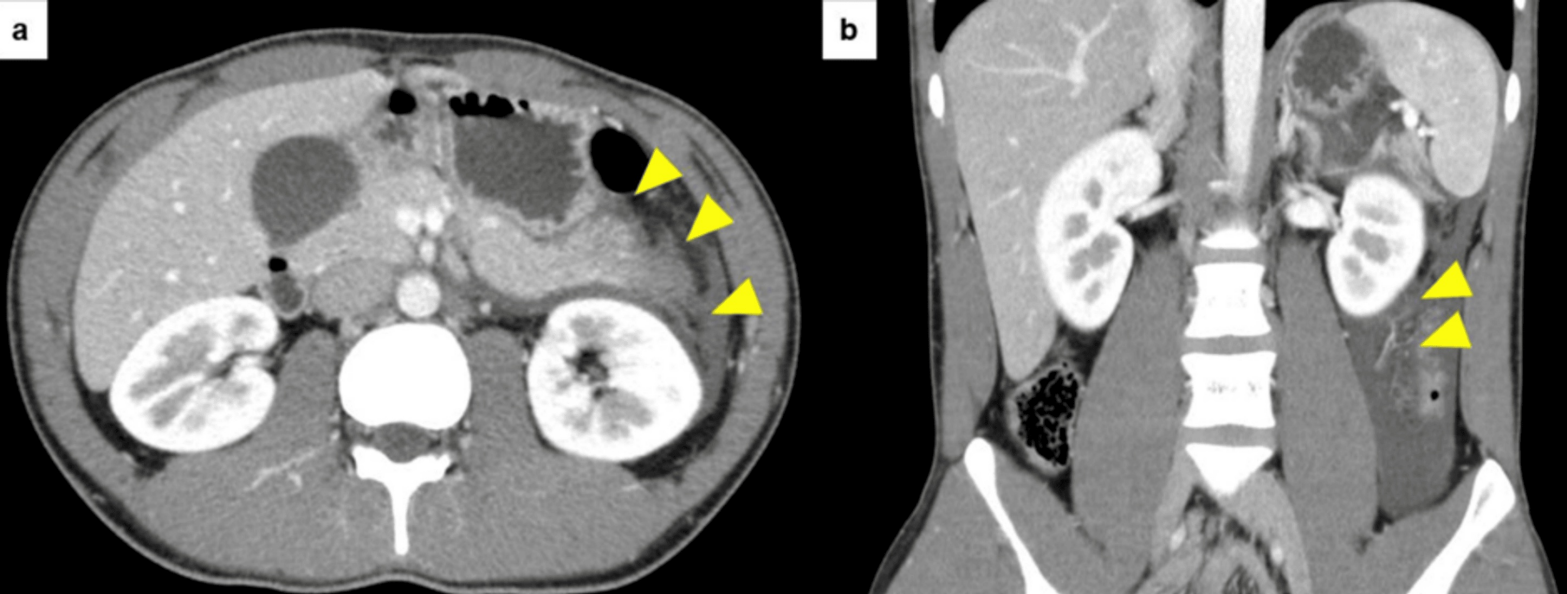
Abdominopelvic contrast-enhanced CT scan on admission. (a) Horizontal section image showing parenchymal enlargement and fluid retention in the pancreatic tail due to inflammation. (b) Coronal section image showing inflammation in the pancreatic tail extending to the lower pole of the kidney. Arrowheads indicate the spread of pancreatitis inflammation.

An initial blood test revealed a white blood cell (WBC) count of 124 × 10²/µL, with 89% neutrophils and a C-reactive protein (CRP) level of 0.29 mg/dL. Additionally, elevated amylase (405 U/L) and lipase (346 U/L) levels were observed (Table 1). However, his liver enzymes and renal function were within the normal ranges. Contrast-enhanced computed tomography (CT) of his abdomen showed an enlarged pancreatic tail parenchyma and hypoabsorptive area and ascites effusion in the lower pole of the kidney (Figures 1a, 1b). Therefore, he was diagnosed with severe AP.

Contrast-enhanced CT and magnetic resonance cholangiopancreatography (MRCP) examinations also showed no anatomical abnormalities or stones in the pancreas or biliary tract, and there was no evidence of malignancy. Other etiologies, such as hypertriglyceridemia (30 mg/dL), hypercalcemia (10.0 mg/dL), and autoimmune diseases (immunoglobulin G4 of 84.2 mg/dL and negative antinuclear antibody) were ruled out (Table 1).

**Table 1:** Laboratory results

	Patient's value	Reference value
Hematology		
White blood cells	124 × 10²/μL	(3,300-8,600)
Hemoglobin	14.9g/dL	(13.7-16.8)
Platelets	28.3×10⁴ /μL	(15.8-34.8)
Blood chemistry		
Amylase	341U/L	(44-132)
Pancreatic amylase	292U/L	(16-52)
Lipase	346IU/L	(11-59)
C-reactive protein	0.29mg/dL	(0-0.14)
Calcium	10.0mg/dL	(8.8-10.1)
Gamma-glutamyl transpeptidase	17IU/L	(13-64)
Total billirubins	0.7mg/dL	(0.4-1.5)
Triglyceride	30mg/dL	(40-234)
Immunoglobulin G4	84.2mg/dL	(11-121)
Antinuclear antibody	<40titer	(<40)

We reviewed the potential infectious causes and ruled them out on the basis of blood-culture test results, and he was negative for serum human immunodeficiency virus. Given that no other drugs had been administered recently, we investigated the relationship between pancreatitis and starting the patient on minocycline. Administration of minocycline was discontinued upon subsequent treatment initiation. After admission, hydration with isotonic crystalloid solution, analgesics, gabexate mesilate, and antibiotics (meropenem), with nothing taken by mouth was initiated.

**Figure 2:**
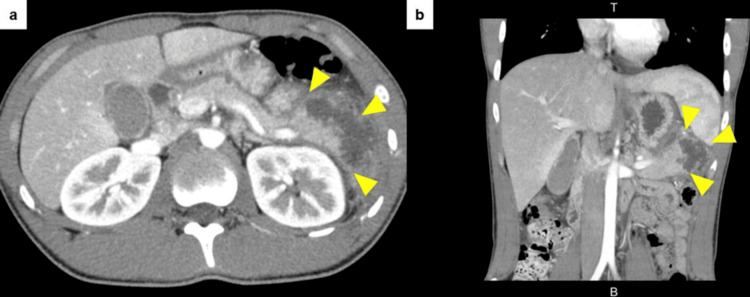
Follow-up CT scan on day 8 of hospitalization. A 4-cm cyst is found in the pancreatic tail. (a) Horizontal section image. (b) Coronal section image. Arrowheads show the extent of the cyst.

**Figure 3:**
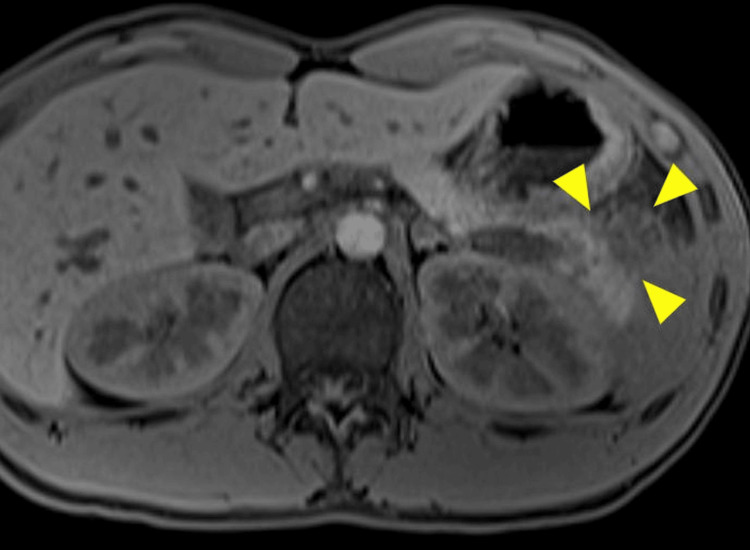
MRCP image at one week after discharging from the hospital. The cyst in the pancreatic tail shrunk to 2 cm. Arrow heads show the extent of the cyst.

From day 2 of administration, the patient developed a fever, and inflammatory markers, such as WBC and CRP, were elevated. However, on day 5 of hospitalization, his fever resolved, and the inflammatory markers showed improvement, so oral intake could be started from day 6 of hospitalization. Follow-up CT on day 8 of hospitalization showed a 4-cm cyst with a homogeneous internal fluid component located in the tail of the pancreas (Figure 2a). The patient was asymptomatic, so we decided to simply observe the cyst. No abdominal pain was observed afterward and no increased inflammatory response was noted in the blood sample test results, so the patient was discharged 14 days after admission. One week after hospital discharge, MRCP showed that the cyst had shrunk to approximately 2 cm (Figure 3). The cyst also was obscured during an endoscopic ultrasound (EUS) examination performed six weeks after hospital discharge. No other abnormal findings in the pancreas were found on EUS. Subsequently, the patient’s condition improved without any recurrence of pancreatitis.

## Discussion

DIAP is a very rare and benign disease. Its incidence is as low as 0.1%-2% of all AP cases [2], and tetracycline-induced AP is limited among them. Although DIAP can cause severe pancreatitis, severe cases are a few. In addition, there have been no reports of tetracycline-induced severe pancreatitis with cyst formation, and we report our experience in this case. Tigecycline, doxycycline, and minocycline have been reported as DIAP with tetracyclines. Among these, DIAP caused by minocycline was reported in 19 cases as shown in Table 2 [5-21]. The severity of the pancreatitis was often mild. There were no cases of cyst formation during the clinical course.

**Table 2:** Reports of tetracycline-induced acute pancreatitis.

Case	Author	Reporting year	Age	Sex	Causal drug	Severity	Number of days administered	Cyst formation
1	Glison et al. [5]	2008	35	M	Tigecycline	Mild	13	-
2	Gabriel et al. [6]	2018	52	M	Tigecycline	Mild	7	-
3	Hung et al. [7]	2009	69	F	Tigecycline	Unknown	7	-
4	Moy and Kapila [8]	2016	51	M	Doxycycline	Mild	3	-
5	Lipshitz et al. [9]	2009	64	F	Tigecycline	Mild	4	-
6	Boyle [10]	2001	29	F	Minocycline	Unclear	10	-
7	Boyle [10]	2001	21	F	Minocycline	Unclear	7	-
8	Marot et al. [11]	2012	64	M	Tigecycline	Mild	3	Unclear
9	Marot et al. [11]	2012	58	M	Tigecycline	Mild	7	Unclear
10	Li and Zheng [12]	2023	75	M	Tigecycline	Unclear	12	-
11	Hemphill and Jones [13]	2016	22	M	Tigecycline	Unclear	10	-
12	Chang et al. [14]	2022	12	F	Tigecycline	Unclear	5	-
13	Albeniz et al. [15]	2017	68	F	Tigecycline	Unclear	5	-
14	Mascarello et al. [16]	2012	Unclear (young)	Unclear	Tigecycline	Severe	12	-
15	Proth-Labarthe et al. [17]	2010	9	M	Tigecycline	Mild	Unclear	-
16	Bassi et al. [18]	2022	65	F	Doxycycline	Mild	3	-
17	Lin et al. [19]	2018	48	F	Tigecycline	Unclear	15	unclear
18	Rawla and Raj [20]	2017	52	F	Doxycycline	Mild	7	-
19	Wang et al. [21]	2021	87	F	Tigecycline	Unclear	6	unclear
20	Ours		17	M	Minocycline	Severe	6	+

According to the Atlanta classification, peripancreatic effusions after the onset of AP are classified as acute peripancreatic fluid collection (APFC), pancreatic pseudocysts, acute pancreatic necrotic collection (ANC) and capsular necrosis (WON), depending on whether the effusion is time-course fluid collection or necrotic collection [22].

Of these, infectious complications due to ANC and WON are among the most common causes of death in AP, so accurate diagnosis and treatment are important [3,22]. The cyst in the present case was considered to be an APFC, as the interior was seen as a uniform liquid component on CT, there was no obvious traffic with the main pancreatic duct on MRCP and the peripancreatic reservoir layer showed gradual shrinkage over time.

Although the peripancreatic reservoir gradually shrank over time, careful follow-up is necessary because the peripancreatic reservoir may become necrotic over time and interventional treatment is required if infectious pancreatic necrosis occurs [3]. This case of pancreatitis with cyst formation is considered a very useful report in the treatment of DIAP.

The mechanisms of DIAP are divided into the following two categories: dose-dependent drug-specific toxicity, as with alcohol, and dose-independent susceptibility, as with antibiotics, which is related to patient susceptibility. The reported mechanism of drug action of tetracyclines is binding to the 30S ribosome. However, the mechanism by which tetracyclines cause pancreatitis remains unclear, although the formation of toxic metabolites, hypertriglyceridemia and high bile acid concentrations are thought to be possible mechanisms [1,23].

## Conclusions

Although AP with minocycline-induced pancreatic cyst formation has not been reported in the past, cyst formation may occur during the clinical course of AP, as in this case. Therefore, careful imaging evaluation and medical treatment are necessary for DIAP as well, and further reports and studies on the local complications of the pancreas are needed.
